# Treadmill exercise within lower body negative pressure protects leg lean tissue mass and extensor strength and endurance during bed rest

**DOI:** 10.14814/phy2.12892

**Published:** 2016-08-05

**Authors:** Suzanne M. Schneider, Stuart M. C. Lee, Alan H. Feiveson, Donald E. Watenpaugh, Brandon R. Macias, Alan R. Hargens

**Affiliations:** ^1^ University of New MexicoAlbuquerqueNew Mexico; ^2^ Wyle Science, Technology, and Engineering GroupHoustonTexas; ^3^ NASA Johnson Space CenterHoustonTexas; ^4^ University of North Texas Health Science CenterFort WorthTexas; ^5^ University of CaliforniaSan DiegoCalifornia

**Keywords:** Body composition, head down tilt, isokinetic, microgravity, muscle atrophy, spaceflight

## Abstract

Leg muscle mass and strength are decreased during reduced activity and non‐weight‐bearing conditions such as bed rest (BR) and spaceflight. Supine treadmill exercise within lower body negative pressure (LBNP_EX_) provides full‐body weight loading during BR and may prevent muscle deconditioning. We hypothesized that a 40‐min interval exercise protocol performed against LBNP_EX_ 6 days week^−1^ would attenuate losses in leg lean mass (LLM), strength, and endurance during 6° head‐down tilt BR, with similar benefits for men and women. Fifteen pairs of healthy monozygous twins (8 male and 7 female pairs) completed 30 days of BR with one sibling of each twin pair assigned randomly as the non‐exercise control (CON) and the other twin as the exercise subject (EX). Before and after BR, LLM and isokinetic leg strength and endurance were measured. Mean knee and ankle extensor and flexor strength and endurance and LLM decreased from pre‐ to post‐BR in the male CON subjects (*P* < 0.01), but knee extensor strength and endurance, ankle extensor strength, and LLM were maintained in the male EX subjects. In contrast, no pre‐ to post‐BR changes were significant in the female subjects, either CON or EX, likely due to their lower pre‐BR values. Importantly, the LBNP_EX_ countermeasure prevents or attenuates declines in LLM as well as extensor leg strength and endurance. Individuals who are stronger, have higher levels of muscular endurance, and/or have greater LLM are likely to experience greater losses during BR than those who are less fit.

## Introduction

Decreased lower body muscle mass, strength, and endurance frequently are observed in non‐weight‐bearing conditions such as limb immobilization, bed rest (BR), and spaceflight (Adams et al. 2003). These skeletal muscle changes are attributed to gravitational unloading, reduced muscle perfusion (Schneider and Convertino 2011), reduced physical activity (Blanc et al. 2000), and during spaceflight to reduced caloric intake (Stein 2001). Baldwin (Baldwin et al. 1996) reviewed the effects of microgravity on the biochemical properties of muscle and suggested that unloading alters the sensitivity of selected muscle fibers to anabolic factors such as growth hormone, insulin‐like growth factor, glucocorticoids, or thyroid hormones, thus reducing protein synthesis and increasing degradation. During and after spaceflight, the ensuing functional reductions in muscle power, strength, and endurance may limit an astronaut's ability to complete tasks or to safely egress the spacecraft in the event of an emergency (Ryder et al. 2013). Muscle weakness also may increase susceptibility to reloading and/or fall‐induced injuries and increase reconditioning time after flight.

The use of exercise countermeasures began early in US and Russian space programs and continues to be a primary method to protect muscle mass and strength. However, exercises employed have not fully prevented muscle atrophy and strength losses during spaceflight, and individual responses are highly variable (Ploutz‐Snyder et al. 2014). Shuttle and the International Space Station aerobic in‐flight exercise hardware provide only partial weight bearing (cycle ergometer or treadmill exercise with the subject tethered to the treadmill using a body harness with bungee cords or a spring‐loaded device) (Cavanagh et al. 2005, 2010). While it is generally accepted that resistive exercise is the most effective approach to maintain muscle function during spaceflight (Baldwin et al. 1996), high‐load resistive exercise devices were not available until the International Space Station (Trappe et al. 2009; Korth 2015; Loehr et al. 2015).

Isokinetic muscle strength testing from both Shuttle and ISS crews suggests that treadmill exercise running provides some level of protection (Trappe et al. 2009; Hayes et al. 2013), but the question remains as to whether the effectiveness of this countermeasure could be enhanced with full body weight or greater loading. With the current harness system, astronauts can typically only tolerate 70–80% of the one body‐weight loading while walking or running on the treadmill. Formulating an effective aerobic exercise protocol which preserves muscle would be critical for astronauts who do not perform high‐intensity resistive exercise, either due to discomfort associated with heavy loads (loading equivalent to body weight plus training load), injury, or failure of the resistive hardware device (Lee et al. 2014; Loehr et al. 2015).

Over the past 2.5 decades, we have developed and tested an exercise device that allows supine walking and running on a vertically oriented treadmill within a lower body negative pressure chamber (LBNP_EX_) (Hargens et al. 1991). We have demonstrated that this device produces footward loading, footward fluid distribution, and cardiovascular responses comparable to those experienced during normal ambulatory conditions in normal Earth gravity (1‐g) (Boda et al. 2000). Furthermore, we have reported that this form of exercise during BR prevents increased bone resorption (Smith et al. 2003; Zwart et al. 2007), maintains aerobic and sprint capacity (Lee et al. 2007, 2009; Schneider et al. 2009), maintains back strength and function (Cao et al. 2005; Macias et al. 2007), and attenuates the decline in orthostatic tolerance (Watenpaugh et al. 2007; Guinet et al. 2009). However, while this countermeasure prevents plantar muscle strength loss during 15 days of BR (Watenpaugh et al. 2000), we had not performed a comprehensive evaluation of the effectiveness of LBNP_EX_ alone to attenuate reductions in leg lean mass (LLM), strength, or endurance during longer duration BR when muscle strength and endurance losses are greater.

Additionally, considering that women comprise a significant proportion (30%) of the active astronaut corps (Harm et al. 2001; Ploutz‐Snyder et al. 2014), it is important to investigate the effects of sex on adaptations to BR and exercise countermeasures. The evidence is conflicting about whether sex differences exist in muscle deconditioning during unloading. Several authors suggest that men have greater deconditioning, perhaps related to larger muscle fibers (Lindboe and Platou 1982; Miles et al. 2005), greater relative number of type II myofibers (Miller et al. 1993), a greater sensitivity to peripheral neuromotor inhibition (Koryak 1999), and/or to effects of male or female hormones to alter protein turnover during unloading (Smith and Mittendorfer 2012). On the other hand, Trappe et al. (2007) report that women have greater losses in muscle mass and strength than men during separate BR protocols. Few studies have directly compared male and female subjects during identical BR conditions (e.g., duration and diet) and while performing the same countermeasures.

The purpose of this study was to evaluate the effectiveness of an LBNP_EX_ protocol for preventing losses in leg lean tissue mass, strength, and endurance during 30 days of 6° head‐down tilt BR, a space flight analog. Male and female monozygous twins were studied before and after bed rest, with one serving as a control and the other performing the LBNP_EX_ countermeasure, to examine specifically the effect of sex on BR‐induced adaptations and exercise countermeasures. We hypothesized that LBNP_EX_ would prevent BR‐induced losses in LLM and in isokinetic muscle strength and endurance, and that the LBNP_EX_ countermeasure would be equally effective for men and women.

## Methods

### Overall protocol

This study was part of a larger investigation of the protective effects of LBNP_EX_ on physiologic adaptations to BR. Specifically, we previously reported results from these subjects in the areas of upright aerobic capacity (Lee et al. 2007, 2009), orthostatic tolerance (Watenpaugh et al. 2007), spine and trunk conditioning (Cao et al. 2005; Macias et al. 2007), renal stone risk (Monga et al. 2006), and bone metabolism (Smith et al. 2003; Zwart et al. 2007). Protocols were reviewed and approved by the Institutional Review Boards of University of California‐San Diego (UCSD) and NASA Johnson Space Center (JSC). Subjects received verbal and written explanation of all procedures and signed statements of informed consent prior to participation. The study protocol has been detailed in previous reports, but is repeated with specific reference to the data described herein.

Fifteen pairs of healthy identical twins (8 male and 7 female) volunteered to participate in this study. Subjects received complete physical examinations from qualified physicians and were admitted to the General Clinical Research Center (GCRC) at UCSD for the duration of the study. They were admitted to the GCRC 6 days prior to the start of BR for a period of ambulatory control during which they underwent familiarization and two pre‐BR testing sessions. Subjects then completed a period of strict 6° head‐down tilt BR for 30 days. Subjects were not allowed to be in upright posture at any time. Urination, defecation, showering, and transport to and from all testing and countermeasure sessions were conducted in head‐down or horizontal posture. Within each twin pair, one twin was randomly assigned (coin flip) to the non‐exercise control (CON) group and the other twin to the exercise countermeasure group (EX). This assignment occurred after completion of all pre‐BR baseline data collection to ensure group assignment could not influence or bias those data. Under the assumption that subjects from the same twin pair are more alike than subjects who are not genetically identical, this partially hierarchical experimental design permits accurate evaluation of the effect of exercise and the interaction of exercise with sex on the pre‐ to post‐BR change, albeit at the expense of having less accurate evaluation of the overall effect of sex (see Statistical analyses).

The GCRC dietary staff prepared a diet consisting of 55% carbohydrate, 15% protein, and 30% fat, and dietary records were maintained to insure consistency for all subjects. Initial caloric consumption was prescribed for each subject using the Harris–Benedict equations, adjusting for self‐reported activity level (correction factor: 1.4–1.5) prior to admission to the GCRC. Thereafter, caloric consumption was adjusted such that subjects maintained their body weight within ±1.0 kg. Body weight was measured in the supine position each morning before breakfast.

### Muscle strength and endurance tests

Leg muscle strength and endurance were tested three times, twice before BR, and once on BR day 28. Pre‐BR sessions were separated by at least 48 h. Muscle strength and endurance tests were conducted while subjects were supine, and all tests were conducted on the right leg. Tests used a standard clinical isokinetic dynamometer (Biodex System 2, Biodex Medical Systems, Inc., Shirley, NY). Range of motion was set for each subject and held constant across testing sessions. Before data collection at each testing speed, subjects completed warm‐up and practice repetitions of extension and flexion through the entire range of motion consisting of five repetitions at approximately 50% of perceived maximal effort followed by two to three repetitions at 100% maximal effort. Subjects rested for at least 2 min between practice repetitions and data collection. Gravity correction was employed to remove the effect of limb and limb adapter weight from the torque measurement.

With the exception that tests were performed while subjects were supine to avoid violation of the in‐bed rest posture, the testing protocols here are similar to those used in previous bed rest studies and in astronauts before and after long‐duration space flight (Lee et al. 2014; English et al. 2015). Isometric knee extensor and flexor strengths were tested at 1.05 rad (60°) of knee flexion from full knee extension. Four maximal efforts were held for 5 sec, separated by 30 sec of rest. Isokinetic extensor and flexor strength of the knee were tested at 1.05 rad•sec^−1^ (60°•sec^−1^) across a range of motion of 0.35 rad to 1.66 rad (20° to 95°) of knee flexion from full extension (0 rad, 0°). Isokinetic extensor and flexor strength of the ankle were tested at 1.05 rad•sec^−1^ (60°•sec^−1^) across a range of motion from 0.35 rad (20°) of dorsiflexion to 0.70 rad (40°) of plantarflexion. The anatomical neutral position was defined as 0 rad (0°). All isokinetic strength tests were performed such that subjects exerted a maximal effort in only one direction within each set of repetitions. At the end of the range of motion, subjects were instructed to relax, and the test operator returned the limb to the starting position. Subjects performed six maximal repetitions of each movement. For the assessment of knee extensor and flexor endurance, subjects performed 21 maximal repetitions in both directions in the same test without pauses or pacing at 2.09 rad•sec^−1^ (120°•sec^−1^) across the same range of motion as the strength tests. Using similar procedures in our laboratory, isokinetic tests have a high degree of reliability (ICC > 0.90), except for ankle dorsiflexion (ICC = 0.67) (Lee et al. 2014).

Peak torque was determined for each repetition of maximal effort during isometric and isokinetic testing. Knee isokinetic endurance was calculated as total amount of work performed for repetitions 2 through 21. Previous experience in our laboratory has shown that subjects do not obtain a maximal effort in the first repetition of knee extension. The summation of work was calculated separately for extension and flexion.

### Lean tissue mass

A whole‐body scan was acquired pre‐BR and on the second day after BR using Dual energy X‐ray absorptiometry (DXA, GE Lunar, Model DPY‐1Q, Madison, WA). Lean mass was determined for each leg from the whole‐body scan, and the results summed across both legs to represent total LLM. Within subjects, the same operator who was blinded to subject group and gender, conducted the pre‐ and post‐BR scans and the same technician calculated LLM. Whole‐body composition results have been reported previously (Lee et al. 2007, 2009).

### Exercise countermeasure

The exercise device used for this study was the same as used in our previous 5‐day (Lee et al. 1997) and 15‐day BR studies (Watenpaugh et al. 2000). The device consists of a partial vacuum chamber in which subjects ran comfortably on a vertically oriented treadmill (PaceMaster SX‐Pro, Aerobics Inc., Little Falls, NJ). The level of LBNP (decompression) and the size of the waist seal aperture were adjusted such that 1.0 times body weight was achieved with a LBNP level of 50–60 mmHg. Thereafter, the level of LBNP was increased during BR across the exercise sessions as tolerated by the subjects with the goal of achieving 1.2 times body weight by the end of the 30 days BR period, but not to exceed 60 mmHg. For the male twins, during the final 2 weeks of BR, six subjects exercised for at least three sessions at 1.05–1.1 BW, and three completed some of their exercise sessions at 1.2 BW. For the female twins, four subjects exercised at least three sessions at 1.05 BW and one subject exercised at 1.1 BW for an entire session.

EX subjects performed 40 min of exercise 6 days week^−1^. Target exercise intensities for this protocol consisted of 7 min at 40% of pre‐BR VO_2_pk, 3 min at 60%, 2 min at 40%, 3 min at 70%, 2 min at 50%, 3 min at 80%, 2 min at 60%, 3 min at 80%, 2 min at 50%, 3 min at 70%, 2 min at 40%, 3 min at 60%, and 5 min at 40%. After completion of the exercise protocol, LBNP was maintained for 5 min while the subjects resisted the suction pressure against the vertical treadmill belt. Pre‐BR, VO_2_pk was measured using a graded treadmill test to volitional fatigue. As previously described (Lee et al. 2007, 2009), the treadmill protocol consisted of 5 min of level walking at 4.8 km h^−1^ (3 mph) and then three, 3‐min stages of increasing exercise intensity (approximately 65%, 75%, and 85% of pre‐bed rest VO_2_peak) at 0% grade. After the third running stage, treadmill speed was held constant, but the grade was increased in increments of 3%•min^−1^ until test termination. VO_2_pk was determined as the average of the highest VO_2_ measured in two consecutive 30‐sec samples.

### Statistical analyses

Commensurate with the partially hierarchical design of the study, in which twin pairs within sex was the whole‐plot factor and exercise/control was the subplot factor within each pair, we used “split‐plot” ANOVA to test for gender effects, exercise effects, and their interaction where the dependent variable was the change from pre‐ to post‐BR in LLM and in each of 10 muscle strength or endurance outcomes. For purposes of these comparisons, “change” was defined as the post‐BR measurement minus the maximum of the two pre‐BR measurements. In this type of ANOVA, the error term for comparing sexes is based on the between‐twin variance, whereas the error term for assessing the effect of exercise and the interaction between exercise and gender is based on the within‐pair variance. As a result, if subjects from the same twin pair are more alike than subjects from different pairs, CON versus EX comparisons would be more powerful in this study design than they would be in a completely randomized study (e.g., 30 subjects without pairing). On the other hand, sex comparisons under this design would have less power. For each outcome, we assessed the efficiency of using twin pairs in the design by a post‐test evaluation of the twin‐pairs‐within‐sex factor. This is essentially a comparison of between‐pair differences to within‐pair differences after the effects of sex and exercise are removed.

For some outcomes, the above ANOVA model was augmented to include the pre‐BR maximum as a covariate (ANCOVA model) to see if the amount of change could be predicted from pre‐BR levels taking exercise into account. The ANCOVA analyses were intended to address the question of whether sex differences in change after BR could be explained by the generally higher strength and endurance levels of the male subjects pre‐BR, with and/or without exercise. However, it is well known that using covariates with substantial measurement errors or nonrepeatability can lead to biased inference (Greene 2000). To prevent this error, we first checked for the exogeneity of the covariates using instrumental variable regression (IVR) with LLM as the instrument. LLM is well suited for IVR as applied here because it is fairly well correlated with all the outcomes studied and also is a very repeatable measurement. However, because of its lower power, we did not use IVR for direct inference on the effect of exercise or sex, but only for checking covariate exogeneity. Thus, after IVR, if the hypothesis of covariate exogeneity was in reasonable doubt (*P* < 0.15) for a given outcome, we did not attempt to adjust for pre‐BR outcome levels, in which case comparisons were made with ANOVA. After all analyses, linear regression models, equivalent to the above ANOVAs were used to estimate means and confidence intervals for several comparisons of interest. All statistical analyses were performed using Stata Version 13.1 software (Statacorp, Inc., College Station, TX).

The reporting level of statistical significance was set at *P *<* *0.05; however, it should be kept in mind that ANOVAs were run for each of 11 outcomes, with three tests per ANOVA (main effects of sex and exercise, plus the interaction). As a result, we recognize that some of the reported effects with *P *<* *0.05 could have arisen by chance. Methods for controlling the family‐wise error rate to 0.05, such as Bonferroni, Sidak, or Hochberg (Hochberg 1988) are conservative and would eliminate consideration of results with *P *>* *0.005, even if applied separately to the 11 tests made for each ANOVA factor. Alternative methods that control the false discovery rate, such as the one proposed by Benjamini et al. (Benjamini et al. 2006), are not feasible because there were not enough tests in our study (33 total) for these methods to be reliable. Therefore, rather than risk missing potentially important effects, we report changes as being “significant” if *P* < 0.05 in the Results section, but leave interpretation to the reader in light of the overall study.

## Results

### Pre‐bed rest comparisons

Anthropometric statistics for pre‐BR age, body mass, height, and aerobic capacity (VO_2_pk) are shown for the CON and EX groups by sex in Table 1. There were no significant differences between twin pairs. Males had higher values for weight, height, and aerobic capacity than females.

**Table 1:** Pre‐bed rest subject anthropometrics (mean ± SD)

	Men		Women	
	Control (*n* = 8)	Exercise (*n* = 8)	Control (*n* = 7)	Exercise (*n* = 7)
Age (years)	27 ± 5	27 ± 5	24 ± 3	24 ± 3
Body mass (kg)^a^	69.4 ± 10.9	66.5 ± 9.5	57.8 ± 11.5	55.3 ± 8.4
Height (cm)^a^	175.7 ± 13.6	173.5 ± 13.0	164.5 ± 10.3	164.5 ± 9.3
VO_2pk_ (mL kg^−1^ min^−1^)^a^	51.9 ± 2.7	52.8 ± 2.7	44.0 ± 1.6	44.4 ± 1.9

a Significant differences between men and women, ANOVA, *P* < 0.05. There are no significant differences between CON and EX groups.

Before BR, there were no significant differences between CON and EX within sex for LLM or any strength measurement. When comparing the pre‐BR values between men and women, the women had significantly (*P* < 0.01 to *P* < 0.03) smaller values of LLM, concentric knee extensor and flexor strength, isometric knee flexor strength, concentric knee flexor endurance, and concentric ankle flexor strength. There were no significant sex differences in the pre‐BR values for isometric knee extensor strength, concentric knee extensor endurance, or concentric ankle flexor strength.

### Effect of bed rest

Mean and standard deviation for each outcome variable are shown for CON and EX groups by sex in Table 2. *P* **‐**values shown for outcomes flagged by asterisks apply to the predicted mean change accounting for the influence of the maximum pre‐BR score as a covariate. Analyses for other outcomes did not use the maximum pre‐BR score as a covariate because it failed the test for exogeneity (additional details provided in the legend for Table 3). Mean extensor (−20% to −10%) and flexor (−13% to −9%) strength and endurance decreased significantly from pre‐ to post‐BR in all measures except for isometric strength in male CON subjects. LBNP_EX_ mitigated knee and ankle extensor strength losses, however, significant decrements in knee (−12% to −10%) and ankle (−7%) strength losses were observed. Neither female group, CON nor EX, experienced significant decreases in any muscle strength or endurance measure during BR. Similarly, mean LLM decreased significantly (−7%) after BR in the male CON subjects, but there was no evidence of a significant change in leg LLM in the male EX subjects, female CON, or female EX subjects.

**Table 2:** Mean (±SD) muscle strength and endurance before (Pre) and after bed rest (Post), the mean percent change (Δ) from pre‐ to post‐bed rest, and the effect of bed rest on each variable within groups

	Measure	Units	Men				Women			
			Pre	Post	Δ	*P*	Pre	Post	Δ	*P*
Control										
Concentric knee extension^a^	Peak Torque	N‐m	189 ± 44	151 ± 37	−20%	**0.0004**	113 ± 31	102 ± 31	−10%	0.22
Concentric knee flexor	Peak Torque	N‐m	88 ± 15	77 ± 13	−13%	**0.0009**	52 ± 12	48 ± 11	−8%	0.11
Isometric knee extension^a^	Peak Torque	N‐m	221 ± 59	198 ± 63	−10%	0.02	147 ± 41	124 ± 34	−16%	0.02
Isometric knee flexion	Peak Torque	N‐m	82 ± 15	76 ± 20	−7%	0.11	49 ± 10	46 ± 10	−6%	0.37
Concentric knee extension^a^	Total Work	N‐m	2458 ± 743	2112 ± 531	−14%	**0.0000**	1442 ± 353	1400 ± 379	−3%	0.36
Concentric knee flexion	Total Work	N‐m	1322 ± 272	1120 ± 198	−15%	**0.0002**	801 ± 179	702 ± 204	−12%	0.03
Concentric ankle extension	Peak Torque	N‐m	94 ± 18	81 ± 26	−14%	**0.0005**	54 ± 14	49 ± 12	−9%	0.09
Concentric ankle flexion	Peak Torque	N‐m	32 ± 7	29 ± 7	−9%	**0.004**	22 ± 4	21 ± 6	−5%	0.10
Leg lean tissue mass	Lean mass	kg	18.5 ± 4.2	17.2 ± 3.2	−7%	**0.0002**	12.4 ± 1.4	12.3 ± 1.3	−1%	0.78
Exercise										
Concentric knee extension^a^	Peak Torque	N‐m	183 ± 48	173 ± 57	−5%	0.21	112 ± 24	104 ± 28	−7%	0.35
Concentric knee flexor	Peak Torque	N‐m	86 ± 24	77 ± 26	−10%	**0.003**	50 ± 11	50 ± 13	0%	0.86
Isometric knee extension^a^	Peak Torque	N‐m	206 ± 55	197 ± 61	−4%	0.28	136 ± 32	121 ± 31	−11%	0.10
Isometric knee flexion	Peak Torque	N‐m	84 ± 25	74 ± 21	−12%	0.01	50 ± 11	45 ± 9	−10%	0.18
Concentric knee extension^a^	Total Work	N‐m	2272 ± 911	2209 ± 978	−3%	0.16	1403 ± 360	1440 ± 398	+3%	0.42
Concentric knee flexion	Total Work	N‐m	1283 ± 488	1123 ± 360	−12%	**0.001**	765 ± 146	705 ± 153	−8%	0.16
Concentric ankle extension	Peak Torque	N‐m	87 ± 23	83 ± 21	−5%	0.27	52 ± 12	55 ± 11	+6%	0.42
Concentric ankle flexion	Peak Torque	N‐m	30 ± 7	28 ± 8	−7%	**0.002**	21 ± 3	20 ± 4	−5%	0.03
Leg lean tissue mass	Lean mass	kg	17.0 ± 3.9	16.5 ± 3.2	−3%	0.10	12.3 ± 1.3	12.5 ± 1.4	+2%	0.42

a Analysis performed with the pre‐bed rest score as a covariate. With Bonferroni adjustment for multiple sampling, a conservative interpretation is that a *P* value of <0.0125 is significant.

**Table 3:** Inference on the effects of sex, exercise, pre‐bed rest score (Pre‐BR), and on the relative efficiency of using matched twins in the design (Twin), on the change score from pre‐ to post‐bed rest. Entries are *P*‐values for testing the null hypothesis of no effect

Outcome	Mode	Sex	Exercise	Exercise × Sex	Pre‐BR^a^	Pre‐BR × Exercise^a^	Twin
Concentric knee extension	Peak Torque	0.86	0.14	0.66	0.64	0.06	0.62
Concentric knee flexor	Peak Torque	0.05	0.36	0.71			0.33
Isometric knee extension	Peak Torque	0.82	0.45	0.66	0.71	0.27	0.56
Isometric knee flexion	Peak Torque	0.47	0.38	0.69			0.10
Concentric knee extension	Total Work	0.75	0.02	0.40	0.41	0.002	0.03
Concentric knee flexion	Total Work	0.24	0.30	0.98			0.004
Concentric ankle extension	Peak Torque	0.28	0.01	0.70			0.01
Concentric ankle flexion	Peak Torque	0.45	0.55	0.97			0.005
Leg lean tissue mass	Lean mass	0.06	0.05	0.34			0.03

a Inference on the effect of pre‐bed rest score as a covariate in explaining pre‐post change is valid only for the most repeatable outcomes (test for exogeneity not rejected; *P *>* *0.15). Others not shown.

### Effect of exercise

**Figure 1:**
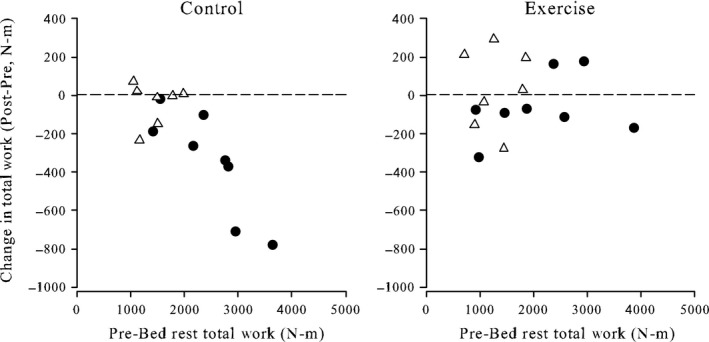
Change in knee extensor muscle endurance (total work) in the control and exercise countermeasure male twins (solid circles) and female twins (open triangles) after 30 days of bed rest.

**Figure 2:**
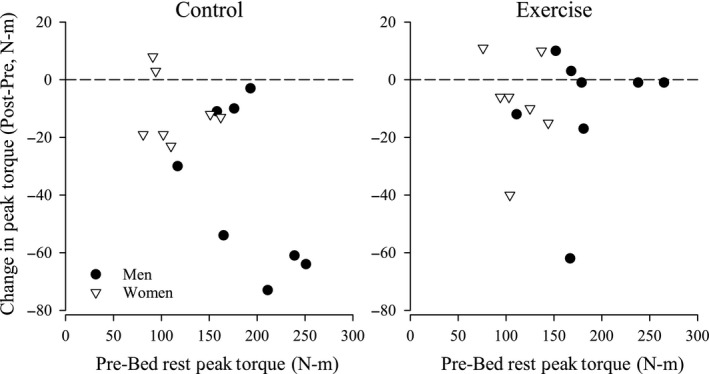
Change in knee extensor strength (peak torque) in the control and exercise countermeasure male twins (sold circles) and female twins (open triangles) after 30 days of bed rest.

For extensor strength and endurance outcomes, we found that the effect of pre‐BR muscle performance on post‐BR performance was different between CON and EX groups. This difference was most evident for knee extensor endurance (*P *=* *0.002, Fig. 1) and less for knee extensor strength (*P *=* *0.063, Fig. 2). For example, CON subjects with higher pre‐BR performance had greater loss in knee extensor endurance than those with lower initial performance. By contrast, EX subjects experienced little discernible effect of pre‐BR performance on change. We were unable to test for this effect for the flexor muscles because of the high variability in the pre‐BR scores.

Using a piece‐wise linear model to plot the CON total work data in Figure 1, with one linear slope for total work less than 2000 N‐m and a second linear slope for values greater than 2000 N‐m, a breakpoint of 1982 N‐m was found with a fairly large 95% confidence interval (1367, 2597), suggesting that the individuals, male or female, with a pre‐BR value below the breakpoint had little change in muscle endurance during BR.

There was no evidence of interaction between sex and exercise on the pre‐ to post‐BR change in any of the muscle strength or endurance measures (Table 3). Similarly, there was no overall effect of sex. We found that the mean loss was significantly less for EX subjects (male and females) only for ankle extensor strength (*P *=* *0.013) and knee extensor endurance (*P *=* *0.023).

**Figure 3:**
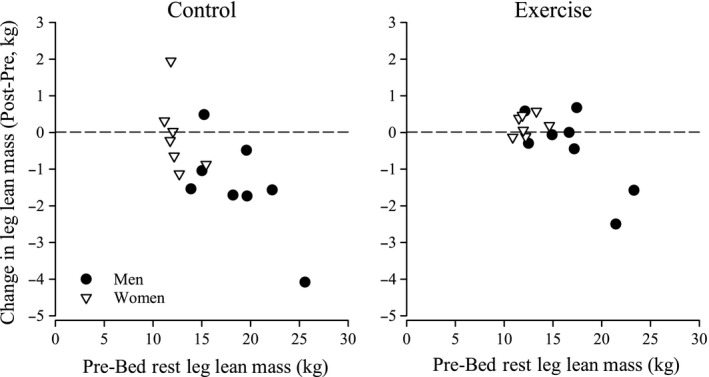
Change in leg lean mass in the control and exercise countermeasure male twins (solid circles) and female twins (open triangles) after 30 days of bed rest.

Without adjusting for pre‐BR LLM, mean LLM tended to decrease as a result of BR to a lesser extent in female subjects (Females: +0.6%, Males: −5%; *P *=* *0.060) and also in EX subjects (EX: −0.9%, CON: −4.7%; *P *=* *0.049, Fig. 3), but there was no evidence of an interaction (*P *=* *0.336) between exercise and sex. In addition, after adjusting for pre‐BR scores, there also was no evidence of either sex or exercise effects on LLM loss.

### Twin‐pair design efficiency

For five of the nine outcomes studied (concentric knee flexion endurance [total work], concentric ankle flexion, concentric ankle extension, LLM, and concentric knee extension endurance [total work]), the split‐plot design using matched twin pairs was more efficient for testing the effect of exercise (*P *<* *0.05) than using unmatched subjects. Conversely, for the other four outcomes, there was no evidence that the split‐plot design was useful for the goal of evaluating exercise.

## Discussion

The question we addressed in this study was whether the losses of LLM, strength and endurance during BR are prevented by an interval‐style treadmill exercise program within LBNP and whether there are sex differences in the BR effects or countermeasure efficacy. This almost daily (6 days week^−1^) moderate aerobic exercise program without resistance exercise prevented the loss of LLM and resulted in smaller decreases in leg extensor strength and endurance during 30 days of BR. Protection of muscle function by an aerobic exercise training program during a model of disuse/unloading has direct implications for astronauts in space, for ambulation‐impaired patients, and for prevention of muscle atrophy in the elderly. In addition, we observed that there is a sex‐specific difference with respect to the BR‐induced loss of strength and LLM; male CON subjects experienced losses while the female CON subjects did not. However, these differences may relate to the pre‐BR levels of muscle fitness, and therefore may not be a true sex effect.

### Ground‐based evidence that aerobic exercise preserves muscle function

Aerobic exercise is not the traditional modality chosen to increase muscle mass or strength. However, the question of whether aerobic exercise offers benefits against muscle deconditioning is a slightly different question that recently has been considered in both the clinical literature and in space flight studies. Harber and coworkers, for example, performed a series of studies to determine whether moderate cycle exercise is sufficient to increase thigh muscle mass in sedentary men (Harber et al. 2012) and women (Harber et al. 2009; Konopka et al. 2010). Their subjects exercised for 12 weeks at 60–80% VO_2_pk, 3–4 days per week. Moderate aerobic exercise increased quadriceps muscle volume and, more specifically, Myosin Heavy Chain1 myofiber cross‐sectional area. Schwartz and coworkers (Schwartz et al. 1991) also reported similar thigh muscle hypertrophy in elderly men during a program of walking and jogging. Harber and coworkers (Harber et al. 2012) considered their findings “particularly important for older individuals, as a single‐mode exercise program increased muscle mass and aerobic capacity, which are two primary risk factors associated with the negative health outcomes of aging”. Thus, developing a training program that is time efficient and not unduly fatiguing would improve adherence.

Few studies exist in which muscle measurements are reported from subjects who perform aerobic exercise to prevent muscle deconditioning during BR or other models of limb unloading. From these studies, we can obtain some insights into the intensity of exercise required to protect muscle function. Suzuki and coworkers (Suzuki et al. 1994) had men and women performing cycle exercise for 1 h day^−1^ at a mild intensity (40% VO_2_pk), and found no benefit from this exercise to preserve leg mass or strength during 10 or 20 days of BR. Sato and coworkers (Sato et al. 2010), on the other hand, employed a similar interval exercise protocol as used in this study during 10 days of unilateral lower limb suspension. This aerobic protocol performed on a cycle ergometer on alternate days maintained thigh muscle volume in 10 male subjects. It is important to note that Suzuki and coworkers employed supine cycle exercise while Sato and coworkers utilized the upright unilateral lower limb suspension model exposing the lower leg to a hydrostatic blood pressure gradient. LBNP_EX_ simulates the upright transmural pressure hydrostatic pressure gradient using vacuum on lower body tissues. Therefore, exercise conducted with vasculature exposed to upright hydrostatic pressure loads may provide an important component to protect against lower limb muscle strength loss.

### Treadmill exercise to maintain muscle function during unloading

In addition to the cardiovascular benefits during spaceflight, treadmill exercise originally was viewed as a method to counteract the changes in lower body muscle mass and strength and to preserve motor coordination (Nicogossian et al. 1994). In the US space program, only passive treadmills were available for the first 10 years of Space Shuttle flights, until a motorized treadmill with a vibration isolation system (TVIS) was deployed in 1999. The motorized mode was postulated to be more effective than the passive mode in maintaining bone and muscle because it allows greater rates of change in force production during walking or running (Davis et al. 1996). However, even with modification of the treadmill harness design to distribute the loading between shoulders and waist more evenly, crewmembers performing treadmill exercise on ISS do not exercise against full‐body loading because of harness discomfort (Cavanagh et al. 2005, 2010; Genc et al. 2006). Genc and coworkers (Genc et al. 2006) speculated that peak forces may be blunted even further during treadmill exercise on ISS by the slight movements of the vibration isolation system, and the forward‐leaning posture (“Groucho walking”) of crewmembers using the harness system. Thus, lower loading forces, altered biomechanics during ambulation, and possible ground reaction force buffering by the isolation system may be responsible for the limited effectiveness of flight treadmill exercise to protect muscle during previous and current space flights.

It is our hypothesis that an LBNP_EX_ device which allows for 100% body weight or greater loading, may provide for better protection of leg muscle mass and strength in microgravity than the current tethered treadmill device with which crewmembers are unable to comfortably exercise with loads approaching full body weight (Cavanagh et al. 2010; Loehr et al. 2015). We previously documented that LBNP_EX_ for only 45 min, 3–4 days week^−1^ together with a flywheel‐resistive exercise protocol maintained aerobic capacity (Schneider et al. 2009) and thigh muscle volume (Trappe et al. 2007) during 60 days of BR in women. LBNP_EX_ allows normal walking and running biomechanics (Boda et al. 2000), without the kinematic changes in gait associated with a tethered treadmill system that reduce peak forces during exercise (McCrory et al. 2002). By adjusting the level of negative pressure and the size of the waist seal opening, we can independently manipulate the degree of cardiovascular strain and musculoskeletal weight bearing. Subjects in the LBNP_EX_ device are able to ambulate comfortably at even greater than one body mass footward loading for at least 45 min without discomfort. If we can maintain full‐body loading and normal biomechanics during exercise in microgravity, this countermeasure should be effective in maintaining skeletal muscle integrity (McCrory et al. 1999).

Bloomfield (Bloomfield 2006) suggests that decreases in lower limb blood flow and perfusion pressure during microgravity reduce the effectiveness of impact loading during exercise to maintain bone mass. The reduction in perfusion and pressure oscillations in the lower body in microgravity are believed to alter the process of mechanotransduction. We propose that by applying LBNP during microgravity exercise, we can restore lower body hemodynamics and tissue fluid pressures to levels similar to upright exercise on Earth and that possible benefits of this form of exercise may protect skeletal and vascular smooth muscle as well as bone mass. In rat hind‐limb suspension studies, the chronic reduction in skeletal muscle blood pressure and flow is associated with reduced metabolic activity and muscle atrophy (Colleran et al. 2000). We also suggest that enhancing muscle hemodynamics during LBNP_EX_ may provide an effective stimulus to restore muscle vascular tone and perfusion, and thus preserve muscle metabolism and the activity of growth factors to preserve muscle mass.

### LBNP_EX_ preserves leg lean mass and strength during BR

Consistent with our hypothesis, our LBNP_EX_ protocol prevents the loss of LLM observed in our CON subjects and in previous BR studies (Convertino 1996; Adams et al. 2003). From our protocol, we cannot conclude whether this effect is due solely to the effective application of body loading during exercise, or whether simulating 1‐g blood hemodynamics and interstitial pressure gradients in the legs contributes to this beneficial response. Decrements in leg muscle strength are consistently reported after 30 days of BR without exercise countermeasures (Convertino 1996; Adams et al. 2003). After about 30 days of BR, decreases in knee and ankle extensor strength are greater than decreases in flexor strength (Convertino 1996). The strength losses in our CON subjects are consistent with this previous literature at our slower testing velocities. However, at the 2.09 rad sec^−1^ testing velocity_,_ extensor and flexor losses were similar. LBNP_EX_ attenuated the decreases in extensor strength and endurance during BR. Interestingly, however, LBNP_EX_ was less effective in attenuating the losses in flexor strength or endurance. This may be due to the lesser degree of deconditioning of the flexor muscles, or to the fact that walking and running movements produce approximately twice the mean muscle force (as a percentage of maximal force) in leg extensor muscles as compared to leg flexor muscles (Besier et al. 2009). In microgravity where extensor movements are reduced or eliminated and extensor muscles are more severely impaired (Baldwin et al. 1996), exercise countermeasures that specifically target the extensor muscles may be required.

### Sex differences in muscle responses during BR

Our results are interesting and potentially controversial, in that we found a greater loss of LLM and muscle strength and endurance during BR in men than in women. We suggest that men in general may have a greater loss of lean tissue mass because of their pre‐BR larger proportion of lean tissue mass and the selective loss of lean tissue versus fat tissue during BR. We previously have reported that the loss of LLM and strength in women who do not exercise during BR is proportional to their pre‐BR condition (Lee et al. 2014).

It is generally thought that women have slower changes in muscle mass in response to training or detraining (Ivey et al. 2000; Mittendorfer and Rennie 2006). A slower training response is often attributed to lower levels of testosterone in women, resulting in a blunted response of muscle protein synthesis in response to overload stimulation (Mittendorfer and Rennie 2006). On the other hand, estrogens exert a protective effect to retain muscle mass in postmenopausal women (Dieli‐Conwright et al. 2009; Messier et al. 2011) and to enhance the recovery of muscle mass in mice after hindlimb suspension (McClung et al. 2006). Another possible reason for differences in the loss of muscle mass with detraining may be sex differences in the type and size of individual muscle fibers. Lindboe and coworkers (Lindboe and Platou 1982) observed that men have a faster rate of thigh muscle atrophy than women after knee surgery. They attributed this to the fact that men have larger and more numerous Type II muscle fibers, which atrophy at a faster rate than Type I myofibers. Women generally have smaller Type I and Type II myofibers (Simoneau and Bouchard 1989; Miller et al. 1993; Staron et al. 2000). Yamamoto and coworkers (Yamamoto et al. 1997) also noted in 11 men and 7 women during 20 days of BR that the rate of loss of leg muscle mass was greater in the men.

The most striking sex difference we observed in muscle function is a lack of loss of leg extensor endurance during BR. Greater muscular endurance in women is frequently noted (Russ and Kent‐Braun 2003; Hunter et al. 2004, 2006; Yoon et al. 2007), even when subjects are matched for strength (Hunter et al. 2004). This endurance difference disappears under conditions of ischemia, suggesting a role for differences in peripheral muscle metabolism or perfusion (Russ and Kent‐Braun 2003). One theory proposes that women's greater endurance is due to a greater proportion of Type I fibers (Simoneau and Bouchard 1989), although this depends on the muscle group studied (Staron et al. 2000). Our study is the first to show that leg extensor endurance is not compromised in women during a 30‐days exposure to BR. However, we believe this is not a true sex effect but rather it is due to the smaller endurance of the women pre‐BR. We determined using a piece‐wise linear model that subjects with a knee extensor endurance less than approximately 1892 N‐m had no significant loss of endurance during bed rest, while those with greater endurance had a decline related to their pre‐BR value. None of the female subjects in this study had an endurance value higher than this value. Only two of the CON males had endurance values below this threshold, perhaps resulting in the greater endurance loss of the male CON group.

### Implications for design of future studies

Hargens and Vico recently commented on the majority of study designs (e.g., different subjects in each group, cross‐over design) that are used in space flight analog studies to examine the effectiveness of countermeasures to deconditioning (Hargens and Vico 2016). The authors suggest that statistical power is reduced when studying unmatched groups with differences in pre‐BR condition and individual variability in the responses to BR. When a cross‐over design is used, an incomplete recovery from a previous BR exposure or repeated exposures to unloading may influence the results. In our analyses, we document that using matched twin pairs is a more efficient way of testing for differences in the majority of muscle outcome measures studied here. From this small group of subjects, it is unclear why the split‐plot design using identical twin subjects was not more efficient for all measures, but the result likely reflects differences within twin pairs due to environmental and behavioral factors as they age; that is, an identical genotype does not imply an identical phenotype. However, planners of future BR studies might consider the split‐plot design, although it is more difficult to find strongly matched subjects (such as twin pairs) willing to undergo BR. Using identical twins exposed to the same BR and testing conditions, preferably with twins participating in BR concurrently, should decrease between subject variability for most outcome measures (Hargens and Vico 2016).

## Conclusions

Based on our results, we suggest that treadmill exercise optimized using LBNP_EX_ is as an important complementary exercise countermeasure to maintain LLM, strength, and endurance during spaceflight in both men and women. Although resistive exercises are more specific and effective for this purpose, some future space flight missions may not permit the currently available high‐intensity resistive exercise platform or there may be individual crewmembers who cannot or should not perform an intense resistive exercise routine. In such scenarios, LBNP_EX_ may maximize the effectiveness of in‐flight or planetary partial‐gravity treadmill exercise by increasing footward loading to the 1‐g levels or greater and perhaps by simulating hemodynamics of 1‐g exercise. Other beneficial features of the LBNP_EX_ countermeasure include its proven effects to protect aerobic capacity (Watenpaugh et al. 2000; Lee et al. 2007, 2009), sprint capacity (Lee et al. 2007, 2009), and bone (Smith et al. 2003; Zwart et al. 2007) and to provide natural gait patterns similar to that of ambulation in 1‐g. Exercise countermeasures to maintain lean body mass and preserve muscle endurance may be especially important for astronauts with higher levels of muscle fitness, given that their level of muscle deconditioning is proportional to their pre‐BR (and pre‐space flight) condition. LBNP_EX_ also may have ground‐based applications to prevent muscle changes with disuse, disease, and aging.
